# Plasma level of alpha-synuclein oligomers as a biomarker for isolated rapid eye movement sleep behavior disorder diagnosis and progression: a prospective cohort study

**DOI:** 10.3389/fneur.2024.1442173

**Published:** 2024-08-23

**Authors:** Chao Ying, Hui Zhang, Ting Wang, Yuan Li, Wei Mao, Songnian Hu, Lifang Zhao, Yanning Cai

**Affiliations:** ^1^Department of Neurobiology, Xuanwu Hospital, Capital Medical University, Beijing, China; ^2^Beijing Geriatric Medical Research Center, Beijing, China; ^3^Key Laboratory for Neurodegenerative Diseases of the Ministry of Education, Beijing Key Laboratory on Parkinson's Disease, Parkinson's Disease Center for Beijing Institute on Brain Disorders, Clinical and Research Center for Parkinson's Disease of Capital Medical University, Beijing, China; ^4^National Clinical Research Center for Geriatric Disorders, Beijing, China; ^5^Department of Neurology, Xuanwu Hospital, Capital Medical University, Beijing, China; ^6^Department of Rehabilitation, Beijing Rehabilitation Hospital, Capital Medical University, Beijing, China; ^7^Department of Clinical Biobank and Central Laboratory, Xuanwu Hospital, Capital Medical University, Beijing, China

**Keywords:** biomarkers, diagnosis, conversion, isolated rapid eye movement sleep behavior disorder, Parkinson’s disease, multiple system atrophy, α-synuclein oligomers

## Abstract

**Background:**

Alpha-synuclein oligomers (o-α-syn) are pivotal in the pathogenesis of α-synucleinopathy. Isolated rapid eye movement (REM) sleep behavior disorder (iRBD) serves as an early indicator of the disease, offering insights into disease mechanisms and early intervention. Nevertheless, the diagnostic and predictive potential of o-α-syn in iRBD remains largely unexplored. This study aimed to evaluate the plasma levels of o-α-syn in patients and investigate their utility as biomarkers for diagnosis of and predicting phenoconversion in iRBD.

**Methods:**

A total of 143 participants, including 77 polysomnography-confirmed iRBD patients and 66 normal controls (NC), were recruited for this longitudinal observational study. Baseline clinical assessments and plasma collection were conducted for all iRBD patients, with 72 of them undergoing regularly prospective follow-up assessments for parkinsonism or dementia. Plasma levels of o-α-syn were quantified using enzyme-linked immunosorbent assay, and were compared between groups using a general linear model adjusted for age and sex. The diagnostic performance of plasma o-α-syn in iRBD was evaluated by area under the receiver operating characteristic curve (AUC) with 95% CI. Cox regression analysis and Kaplan–Meier survival curves were employed to assess the predictive value of plasma o-α-syn for phenoconversion in iRBD.

**Results:**

Plasma o-α-syn levels did not exhibit statistically significant differences among iRBD converter patients, iRBD nonconverter patients, and NC. The AUC for distinguishing NC from iRBD was 0.52 (95% CI: 0.42–0.62, *p* = 0.682). Spearman correlation analysis revealed a significant positive correlation between plasma o-α-syn levels and MOCA scores in the iRBD group (*p* < 0.001). Subgroup analyses indicated that iRBD patients with cognitive decline (*p* = 0.058) and depressive symptoms (*p* = 0.017) had notably lower o-α-syn levels compared to those without such symptoms. Over a median follow-up period of 5.83 years, 26 iRBD patients developed neurodegenerative synucleinopathies. Cox regression and Kaplan–Meier survival curve analyses indicated that plasma level of o-α-syn lacked a predictive value for disease conversion in iRBD patients.

**Conclusion:**

Despite a potential role in the pathophysiology of iRBD, o-α-syn are not appropriate biomarkers for diagnosing or predicting disease progression. While this study offers insights into the pathogenesis of iRBD and neurodegenerative synucleinopathies, further large-scale longitudinal studies are warranted to validate these findings.

## Introduction

Isolated rapid eye movement (REM) sleep behavior disorder (iRBD) is a form of parasomnia characterized by the absence of normal skeletal muscle atonia during REM sleep and accompanied by dream-enacting behaviors, with an estimated prevalence of approximately 1–4% in general population (1). It could significantly impact the quality of life and safety of both patients and their families (2). Numerous longitudinal studies have confirmed that about 80% of iRBD patients will develop α-synucleinopathies such as Parkinson’s disease (PD), Multiple system atrophy (MSA), or Dementia with Lewy bodies (DLB) after several years (3). Therefore, iRBD may represent a highly specific preclinical phase of alpha-synucleinopathies. Further research and exploration of iRBD may help in predicting and intervening in the development of synucleinopathies.

α-synuclein (α-syn) is closely associated with the pathogenesis and progression of synucleinopathies. The characteristic pathological change of PD is the appearance of Lewy bodies and Lewy neurites, which deposit in the cell body and projection of neurons. In contrast, the hallmark pathological structure of MSA is glial cytoplasmic inclusions that accumulate in the cytoplasm of oligodendrocytes. These pathological structures share a common feature of being primarily composed of aggregated α-syn (4). α-syn can self-aggregate into soluble oligomers, protofibrils, or fibrillar structures (5). Oligomers formed by abnormal aggregation of α-syn possess neurotoxic effects, causing weakened axonal transport, induction of mitochondrial dysfunction, and reduced proteasomal activity, ultimately resulting in apoptosis of dopamine neurons (6). Autopsy analyses and animal experiments have shown that oligomeric α-syn is the truly neurotoxic α-syn, and it has been demonstrated that oligomeric α-syn is an important form for the formation of α-syn protofibrils (7). Therefore, α-synuclein oligomer (o-α-syn) may be a potential research target for synucleinopathies.

Pathological changes in α-syn in the peripheral nervous system of patients with α-syn spectrum disorders may precede those in the central nervous system, which opens up the possibility of detecting pathological α-syn in peripheral tissues to enable early diagnosis of α-syn spectrum disorders (8). Currently, there is limited knowledge regarding the exploration of plasma o-α-syn in patients with prodromal synucleinopathies (9). To investigate the diagnostic and predictive ability of o-α-syn in iRBD patients for disease progression toward synucleinopathies is of great significance for elucidating the pathogenesis and providing new insights into their early diagnosis and treatment. Therefore, the present study first assessed the differences in plasma o-α-syn levels between patients with iRBD and normal controls (NC) and prospectively followed up patients with iRBD in order to investigate the potential of baseline levels of plasma o-α-syn in predicting the phenotypic transition of iRBD to synucleinopathy.

## Materials and methods

### Participants

iRBD patients aged >50 years were continuously recruited from and followed up at the Department of Neurology at Xuanwu Hospital, Capital Medical University between October 2012 and June 2017, as described in previous articles by our team (3). The diagnosis of iRBD was confirmed through video polysomnography (vPSG) performed at the sleep laboratory in Xuanwu Hospital, in accordance with the criteria outlined in the International Classification of Sleep Disorders (ICSD-III) (10). Patients with secondary iRBD associated with neurodegenerative diseases, narcolepsy, structural brainstem damage, or those using antidepressants were excluded from the study. All participants underwent neurological evaluations to exclude any symptom or sign of dementia or parkinsonism. Age-and sex-matched NC from the community were evaluated using clinical interviews and the RBD Questionnaire Hong Kong (RBDQ-HK, total score < 18) to confirm the absence of RBD symptoms. All participants underwent neurological evaluations for dementia or parkinsonism at enrollment and follow-up. In the present study, 77 iRBD patients and 66 NC were included. All the included subjects were free of autoimmune diseases and immunosuppressive drug use. The study was approved by the Institutional Review Board and Ethics Committees of the participating hospitals. Written informed consent was obtained from each participant or their legal guardians before inclusion in the study.

### Baseline clinical assessments

Demographic information, such as age, sex, was collected for all participants. Baseline assessments of both motor and non-motor symptoms were conducted for iRBD patients. The severity of RBD symptoms was evaluated using the RBDQ-HK. Motor symptoms were assessed using Part 3 of the Movement Disorder Society-Unified Parkinson’s Disease Rating Scale (MDS-UPDRS). Quantitative motor testing was performed using the Purdue Pegboard. Cognitive function was assessed using both the Montreal Cognitive Assessment (MoCA) scale and the Mini-Mental State Examination (MMSE) to evaluate the overall cognitive status. Cognitive decline was defined as the MoCA score adjusted by educational levels < 26; We used Hamilton Depression Rating Scale (HAMD) and the 30-item Geriatric Depression Scale (GDS-30) to assess the mood state and depression was defined as a score of HAMD ≥8. Constipation was defined using the Rome III criteria (11). Excessive daytime sleepiness (EDS) was evaluated using the Epworth Sleepiness Scale (ESS), with EDS defined as an ESS score ≥ 10. Olfactory function was evaluated using the Brief Smell Identification Test (B-SIT) with hyposmia defined as a score < 8. Orthostatic hypotension (OH) was defined as a decrease in systolic blood pressure of more than 20 mmHg or a diastolic blood pressure drop of over 10 mmHg within three minutes of changing from a lying to a standing position.

### Follow-up visit

We prospectively followed the patients with iRBD through on-site evaluations performed by experienced neurologists specializing in movement disorders, with the goal of diagnosing phenotypic transitions toward parkinsonism or dementia. All patients with iRBD were followed annually or whenever they reported complaints of cognitive, motor, or autonomic function. Full clinical assessments were performed in each follow-up visit. Phenoconversion was noted upon the occurrence of parkinsonism or dementia. We recorded the dates of the last visit and the final diagnosis of neurodegenerative disease for each patient. In this study, we excluded new-onset mild cognitive impairment from being considered a phenotypic transformation, focusing specifically on the conversion of iRBD patients to PD, MSA, or DLB. The clinical diagnosis of PD or MSA was made based on the widely recognized diagnostic criteria established by the Movement Disorder Society and Gilman et al. (12, 13). For patients who progressed to dementia, a potential diagnosis of DLB was made based on the 2017 diagnostic criteria (14). In our cohort, 5 patients with iRBD were lost to follow-up, and 72 patients were finally enrolled in this study. After a mean follow-up period of 5.83 years, our study found that 26 out of 72 patients with iRBD developed definite α-synucleinopathy (the “converted” population), while the remaining 46 patients showed no signs of α-synucleinopathy (the “nonconverted” population).

### Measurement of o-α-syn in the plasma

Prior to baseline venous blood collection, all participants fasted for at least 12 h. Blood samples, collected in 10 mL EDTA-K_2_ anticoagulant tubes, were kept between 2 and 8°C for a maximum of 30 min. The blood samples were centrifuged at 2500 g for 15 min in a pre-cooled centrifuge set at 4°C. After centrifugation, the plasma was mixed thoroughly, aliquoted, and stored at −80°C until further analysis. As described in the previous study, the plasma o-α-syn levels was accurately measured using the HUMAN α-Synuclein PATHO ELISA kit (847–010400108, AnalytikJena, AJ Roboscreen GmbH, Leipzig, Germany) according to the manufacturer’s instructions (15, 16). Calibration curves for plasma o-α-syn levels were established and determined using Discovery Workbench software (version 4.0.12; Meso Diagnostics, LLC). Calibration curves were computed using a four-parameter logistic curve-fitting regression with a weighting factor of 1/Y2. Values below the detection limit of the analytical method were designated as “not detected” and were calculated as half of the lowest detection limit for statistical analyses (17).

### Statistical analysis

The data were analyzed using SPSS 26.0 and R software (version 4.2.1). Quantitative demographic data were assessed for normality using the Shapiro–Wilk test. Normally distributed quantitative data were presented as mean ± SD, while non-normally distributed data were presented as median with interquartile range (IQR). Quantitative data was presented as *n* (%). The t student’s test was utilized to compare the age between the iRBD and NC groups, while the Mann–Whitney U test was employed to compare plasma o-α-syn levels between the iRBD and NC groups. The chi-square test was applied to assess gender differences between the iRBD and NC groups. Furthermore, differences in plasma o-α-syn levels among the NC group, the iRBD-nonconverter group, and the iRBD-converter group were analyzed using a general linear regression model with adjustment for confounding factors (age and sex). ROC curves were generated by bootstrapping 1,000 replicates using the boot.roc algorithm in the fbroc R package to evaluate the diagnostic accuracy of plasma o-α-syn for iRBD. The receiver Operating Characteristic curve (AUC) and 95% confidence interval (CI) was computed. Spearman correlation analysis was conducted to investigate the relationship between plasma o-α-syn levels and clinical variables with false discovery rate (FDR) correction. To examine if baseline markers served as risk biomarkers for the early phenoconversion to neurodegenerative diseases in iRBD patients, plasma o-α-syn were evaluated using Cox regression, adjusting for age and sex to calculate hazard ratios (HRs). Additionally, a Kaplan–Meier survival curve was constructed. For stratification, we determined the optimal cut-off value for o-α-syn in plasma using the X-tile software to stratify iRBD patients into low (< 8.69 pg./mL) and high (≥ 8.69 pg./mL) levels groups. Statistical significance was defined as *p* < 0.05 for all two-tailed tests.

## Results

### Participant characteristics

Table 1 summarizes the baseline demographic and clinical characteristics of all subjects. The iRBD group and the control group showed similar demographic profiles regarding age and sex. RBDQ-HQ scores were significantly higher in the iRBD group compared with the NC group, while MMSE and MOCA scores were significantly lower. There was no statistically significant difference in the plasma o-α-syn levels and detection rate between the iRBD and NC groups. Additionally, Of the 72 iRBD patients, 26 (36.11%) developed a neurodegenerative disease over a median follow-up period of 5.95 years (mean ± SD: 5.83 ± 2.88 years), with an average duration of 4.58 ± 2.51 years from baseline assessment to disease phenoconversion.

**Table 1:** Demographic and clinical characteristics of all subjects at baseline.

Variables	NC (*n* = 66)	iRBD (*n* = 77)	*p*
Age, years	66.84 ± 7.17	67.43 ± 7.35	0.630
Male, *n* (%)	51 (77.27%)	60 (77.92%)	0.926
α-Synuclein oligomers			
Detection rate, *n* (%)	40 (60.61%)	57 (74.03%)	0.087
Concentration, pg./mL	8.33 (2.50, 28.93)	10.45 (2.50, 16.60)	0.673
RBDQ-HK score	1.00 (0.00, 3.00)	60.00 (47.00, 68.00)	< 0.001
MMSE score	29.00 (28.00, 30.00)	28.00 (27.00, 29.00)	0.005
MOCA score	27.00 (25.00, 28.00)	25.00 (23.00, 27.00)	< 0.001
Purdue Pegboard test			
Dominant hand score		13.64 ± 1.61	
Non-dominant hand score		13.04 ± 1.63	
RBD symptom duration, years		3.00 (5.00, 11.00)	
UPDRS-III score		2.00 (0.00, 3.00)	
B-SIT score		7.00 (5.00, 9.00)	
ESS score		6.00 (3.00, 8.00)	
HAMD score		2.00 (1.00, 6.00)	
GDS-30 score		7.00 (4.00, 12.00)	

Continuous variables are reported as mean ± SD or median (IQR), and categorical variables are displayed as numbers (%). NC, Normal Control; RBD, Rapid Eye Movement Sleep Behavior Disorder; iRBD, isolated Rapid Eye Movement Sleep Behavior Disorder; RBDQ-HK, Rapid Eye Movement Sleep Behavior Disorder Questionnaire-Hong Kong; UPDRS-III, Unified Parkinson’s Disease Rating Scale part III; MMSE, Mini Mental Status Examination; MoCA, Montreal Cognitive Assessment; HAMD, Hamilton Depression Rating Scale; ESS, Epworth Sleepiness Scale; OH, orthostatic hypotension; GDS, Geriatric Depression Scale; B-SIT, Brief Smell Identification Test.

### Plasma o-α-syn levels in iRBD and NC

**Figure 1:**
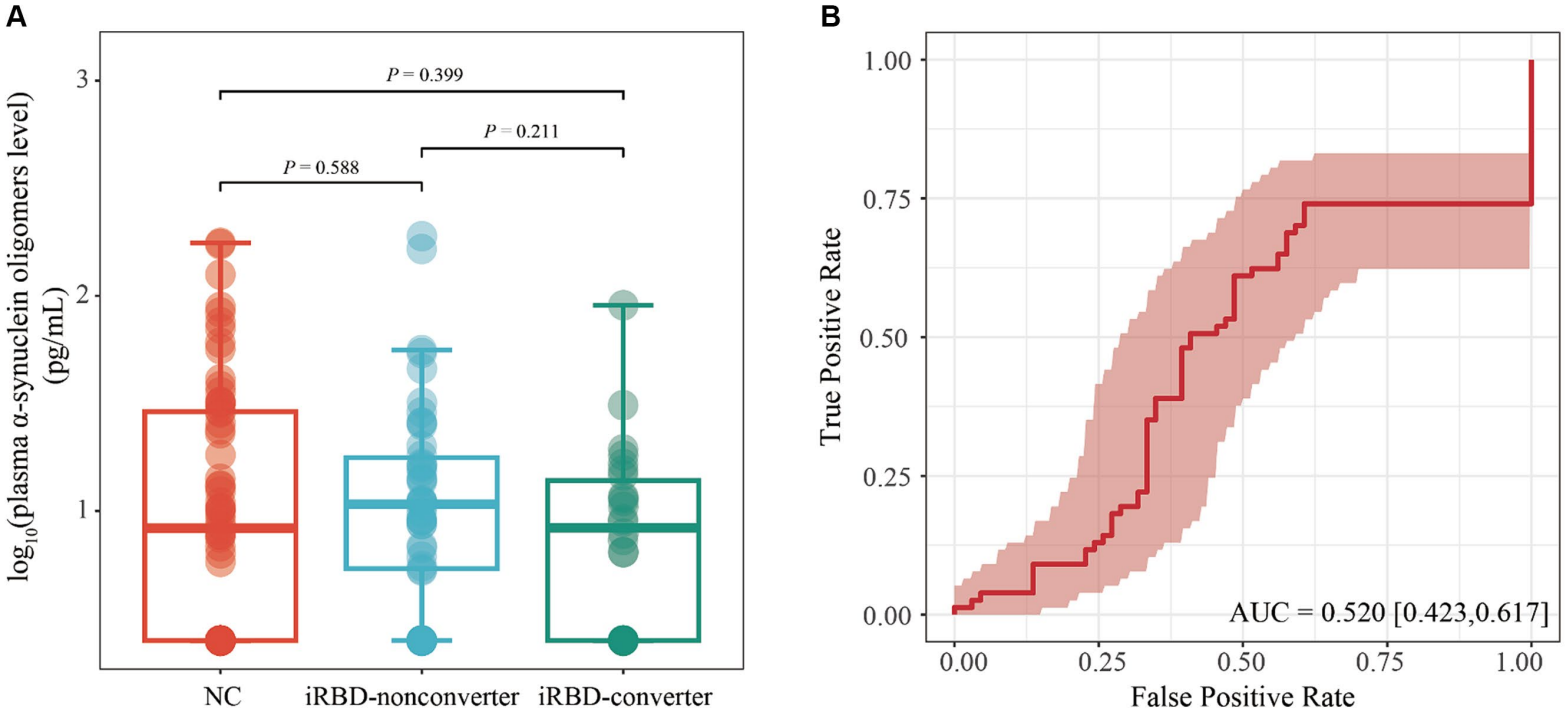
Performance of plasma level of α-synuclein oligomers in diagnosing iRBD. **(A)** Comparison of plasma levels of α-synuclein oligomers among NC, iRBD-nonconverter, and iRBD-converter patients. Statistical comparisons were conducted using a generalized linear model with adjustments for age, gender. **(B)** ROC evaluated the ability of plasma level of α-synuclein oligomers to distinguish patients with iRBD from NC. iRBD, Idiopathic Rapid Eye Movement Sleep Behavior Disorder; NC, Normal Control; AUC, Area Under the Curve; ROC, Receiver operating characteristic; CI, Confidence interval.

We utilized a general linear model to evaluate disparities in plasma o-α-syn among the NC, iRBD-nonconverter, and iRBD-converter groups with adjustment for age and sex. Our results demonstrated no statistically significant variations in plasma o-α-syn among the three groups (iRBD-nonconverter group vs. NC, *p* = 0.588; iRBD-converter group vs. NC, *p* = 0.399; RBD-nonconverter vs. iRBD-converter group, *p* = 0.211) (Figure 1A). ROC curve analysis indicated that plasma o-α-syn levels had limited diagnostic value in discriminating iRBD from NC, with AUC of 0.520 (95% CI: 0.423–0.617, *p* = 0.665), sensitivity of 0.740 (95% CI: 0.642–0.838), and specificity of 0.394 (95% CI: 0276–0.512) (Figure 1B).

### Correlation of plasma o-α-syn with clinical characteristics in iRBD

Employing Spearman correlation analysis with FDR correction, we then investigated the association between plasma o-α-syn and clinical characteristics within the iRBD group, as outlined in Table 2. We found a significant positive correlation between plasma o-α-syn and MoCA scores in all iRBD patients (*Rho* = 0.386, FDR-adjusted *p* = 0.006). However, this correlation was not present in either the iRBD-nonconverter or iRBD-converter groups. Moreover, plasma o-α-syn levels was correlated with several factors, such as baseline age, duration of RBD symptoms, RBDQ-HK score, UPDRS-III score, MMSE score, ESS score, HAMD score, GDS-30 score, Purdue Pegboard test, and B-SIT score, among all iRBD patients, as well as within the iRBD-nonconverter and iRBD-converter groups.

**Table 2:** Spearman correlation between clinical characteristics and plasma α-synuclein oligomers levels in patients with iRBD.

	All iRBD patients			iRBD non-converter			iRBD converter		
	Rho	*p*	FDR adjusted *p*	Rho	*p*	FDR adjusted *p*	Rho	*p*	FDR adjusted *p*
Age at baseline	−0.080	0.343	0.457	−0.181	0.230	0.369	−0.316	0.116	0.453
RBD symptom duration	−0.267	0.023	0.072	−0.175	0.246	0.369	−0.424	0.031	0.372
RBDQ-HK score	−0.260	0.022	0.072	−0.242	0.106	0.258	−0.153	0.457	0.649
UPDRS-III score	−0.252	0.027	0.072	−0.297	0.045	0.180	−0.120	0.560	0.668
MMSE score	0.164	0.153	0.262	0.227	0.129	0.258	−0.064	0.757	0.757
MoCA score	0.386	0.001	**0.006**	0.378	0.010	0.090	0.300	0.136	0.453
ESS score	−0.007	0.951	0.951	−0.152	0.314	0.409	0.143	0.485	0.649
HAMD score	−0.059	0.608	0.663	0.084	0.577	0.629	−0.169	0.411	0.649
GDS-30 score	−0.099	0.394	0.473	−0.010	0.949	0.949	−0.143	0.487	0.649
B-SIT score	0.118	0.309	0.457	0.144	0.341	0.409	0.266	0.190	0.456
Dominant hand score*	0.184	0.109	0.218	0.356	0.015	0.090	−0.104	0.612	0.668
Non-dominant hand score*	0.247	0.030	0.072	0.227	0.129	0.258	0.290	0.151	0.453

*From Purdue Pegboard test. Bold values: FDR adjusted *p* < 0.05. RBD, Rapid Eye Movement Sleep Behavior Disorder; iRBD, isolated Rapid Eye Movement Sleep Behavior Disorder; RBDQ-HK, Rapid Eye Movement Sleep Behavior Disorder Questionnaire-Hong Kong; UPDRS-III, Unified Parkinson’s Disease Rating Scale part III; MMSE, Mini Mental Status Examination; MoCA, Montreal Cognitive Assessment; HAMD, Hamilton Depression Rating Scale; ESS, Epworth Sleepiness Scale; GDS, Geriatric Depression Scale; B-SIT, Brief Smell Identification Test.

### Comparison of plasma o-α-syn levels in iRBD patients with different non-motor subtypes

Subgroup analyses revealed a trend indicating lower plasma o-α-syn levels in iRBD patients with cognitive impairment and depression compared to those without these symptoms (adjusted *p* = 0.058). Furthermore, iRBD patients with depressive symptoms exhibited significantly lower plasma o-α-syn levels compared to those without (adjusted *p* = 0.017). Conversely, subgroup analyses revealed no statistical differences between the two groups regarding non-motor symptoms like hyposmia, excessive daytime sleepiness, constipation, and OH (Table 3).

**Table 3:** Comparison of α-synuclein oligomer levels in iRBD patients with different non-motor subtypes.

Symptom	Subgroup	α-synuclein oligomer level (pg/ml)	p*
Cognitive decline	YES (*n* = 47)	16.587 ± 35.025	0.058
	NO (*n* = 30)	20.547 ± 20.150	
Hyposmia	YES (*n* = 45)	13.257 ± 15.999	0.240
	NO (*n* = 32)	24.983 ± 41.990	
Depression	YES (*n* = 14)	7.324 ± 5.957	**0.017**
	NO (*n* = 63)	20.532 ± 32.635	
Excessive daytime sleepiness	YES (*n* = 12)	25.719 ± 52.096	0.504
	NO (*n* = 65)	16.729 ± 24.309	
Orthostatic hypotension	YES (*n* = 29)	17.567 ± 34.723	0.473
	NO (*n* = 48)	18.470 ± 27.179	
Constipation	YES (*n* = 55)	17.034 ± 25.989	0.406
	NO (*n* = 22)	20.870 ± 38.906	

*Covariance analysis adjusted for age and sex. Bold values: *p* < 0.05. Data are expressed as mean ± SD.

### Plasma o-α-syn levels at baseline to predict iRBD conversion

**Figure 2:**
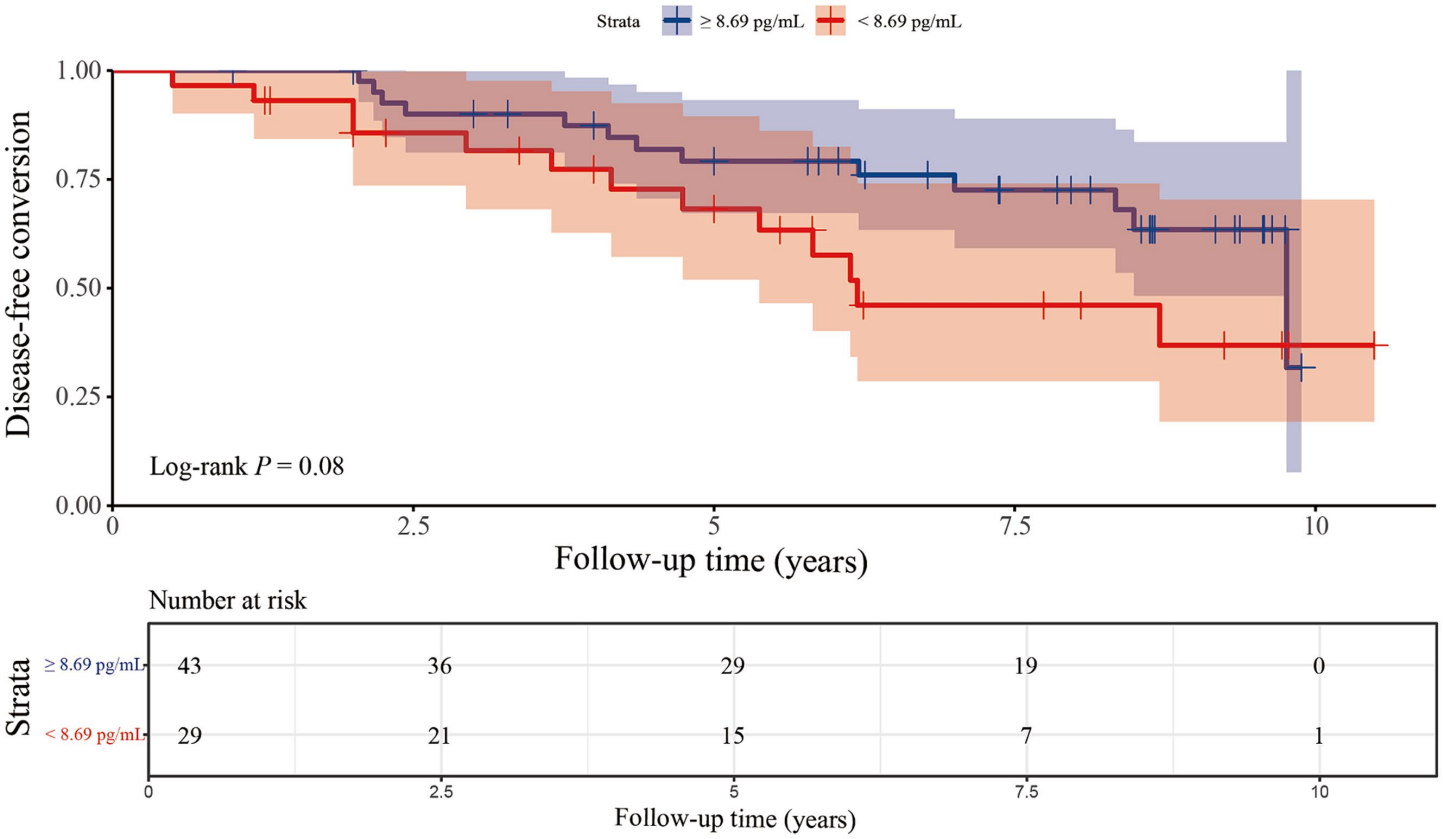
Kaplan–Meier plots of disease-free survival of patients with iRBD stratified according to the level of plasma α-synuclein oligomers. The plasma levels of α-synuclein oligomers were categorized into higher-and lower-level groups using a cut-off point of 8.69 pg./mL.

Kaplan–Meier survival curves were drawn according to the cut-off value of the o-α-syn levels (Figure 2). Disease-free conversion of iRBD was not higher in patients with the o-α-syn levels under the cut-off value (log-rank test, *p* = 0.080). Cox regression analysis was performed to evaluate the predictive value of baseline plasma o-α-syn levels for disease progression from iRBD to α-synucleinopathy. After adjusting for age and sex, per SD increase in baseline plasma o-α-syn levels did not predict the development of neurodegenerative synucleinopathies in iRBD patients (adjusted HR: 0.638, 95% CI: 0.293–1.388, *p* = 0.257). Compared to patients with higher o-α-syn levels, the risk of disease-free conversion for iRBD patients was 2.012 times higher for individuals with a low levels of o-α-syn, while the *p*-value from the Log-rank test only reached borderline significance (adjusted HR = 2.012, 95% CI: 0.923–4.386, *p* = 0.079). Stratifying individuals into three tertiles based on plasma o-α-syn levels, comparison between individuals in the highest tertile and those in the lowest tertile at baseline did not show a significant reduction in hazard ratio (adjusted HR = 0.537, 95% CI: 0.201–1.438, *p* = 0.216). Moreover, there was no significant dose–response relationship between plasma o-α-syn levels and the risk of conversion in iRBD patients (*P*_trend_ = 0.222), as shown in Table 4.

**Table 4:** Predictors of conversion in iRBD patients in COX regression analysis based on plasma α-synuclein oligomers level.

α-synuclein oligomers	Model 1			Model 2		
	HR	95% CI	*p*	HR	95% CI	*p*
Per SD	0.641	0.298–1.376	0.253	0.638	0.293–1.388	0.257
≥ 8.69 pg./mL	1.000	Reference	-	1.000	Reference	-
< 8.69 pg./mL	1.975	0.910–4.288	0.085	2.012	0.923–4.386	0.079
1st tertile	1.000	Reference	-	1.000	Reference	-
2nd tertile	0.800	0.325–1.971	0.627	0.766	0.303–1.935	0.573
3rd tertile	0.549	0.207–1.460	0.230	0.537	0.201–1.438	0.216
P_trend_			0.231			0.222

Model1: Crude; Model2: adjusted for age and sex. iRBD, isolated Rapid Eye Movement Sleep Behavior Disorder; HR, Hazard Ratio; CI, Confidence Interval; −, Not available.

## Discussion

In this longitudinal study, we examined plasma o-α-syn as diagnostic and predictive markers for the progression of iRBD to synucleinopathies, such as PD, DLB, and MSA, while also assessing their correlations with clinical variables. Our study firstly found no significant differences in baseline plasma o-α-syn levels among the iRBD-converter group, iRBD-nonconverter group, and NC group. Similarly, plasma o-α-syn levels were ineffective in distinguishing between iRBD patients and the control group. However, we found a significant positive correlation between plasma o-α-syn levels and MoCA scores in iRBD patients. Furthermore, during follow-up, we did not observe that baseline plasma o-α-syn levels were associated with the risk of conversion to synucleinopathy in patients with iRBD.

Although the mechanism of action of pathological α-syn in neurodegenerative diseases is currently unclear, the prevailing view is that it is α-syn aggregates (mainly oligomers), rather than phosphorylated α-syn, that are involved in the pathogenesis of α-syn spectrum diseases (18). It is believed that oligomeric α-syn exerts its neurotoxic effects mainly through mitochondrial functional impairment, defective endoplasmic reticulum action, protease action, glial cell inflammatory response, cell membrane damage, defective lysosomal function and synaptic dysfunction (7). From the few studies analyzing cerebrospinal fluid (CSF) biomarkers in patients with iRBD, RT-QuIC detected pathogenic α-syn with a sensitivity of 90–100% and a specificity of 90–98%, with positive results suggesting an increased risk of phenotypic switching, highlighting the presence of pathogenic α-syn (including o-α-syn) at the iRBD stage and that this misfolded α-syn is associated with disease diagnosis and prognosis (19). While plasma o-α-syn may serve as potential biomarkers for synucleinopathies, their performance during the prodromal phase remains unclear. Our results demonstrate that plasma levels of o-α-syn in the iRBD patients showed no significant difference compared to NC, which is consistent with previous research findings. Compta et al. (20) found no significant difference in o-α-syn levels in the CSF of iRBD patients compared to NC, while PD patients exhibited significantly higher levels of o-α-syn in CSF compared to the control group. Park et al. (21) similarly found significantly higher levels of o-α-syn in the CSF of PD patients compared to controls, but observed no significant difference between the two groups in plasma o-α-syn levels. Interestingly, Fouldset al (22). investigated plasma levels of α-syn in 32 PD patients and 30 controls, revealing significantly higher levels of phosphorylated α-syn in the PD group compared to controls, but no significant differences in total α-syn or o-α-syn levels between the two groups. This finding in plasma was corroborated by the Pchelina team, who likewise detected no significant difference in plasma o-α-syn levels between PD and NC (23). However, several studies also support an increase of o-α-syn levels in PD patients in plasma, saliva, or CSF (Table 5) (24–28). In each study, there was significant overlap in α-syn levels between patients with IRBD or PD and controls (19). These diverse results may be attributed to factors including hemolysis-induced red blood cell α-syn contamination, variations in internal development and commercially available immunochemical methods, types of calibrators used to establish standard curves, and differences in detection/quantification methods, as well as potential biases in analytical factors, sample size, racial characteristics, and selection biases (29, 30). Therefore, our research findings need further confirmation from larger cohorts and independent laboratories.

**Table 5:** Summary of studies on alpha-synuclein oligomers in body fluids included in the discussion section.

Study	Publication year	Biofluid sample	Study group	Detection methods	Results
Compta et al. (20)	2015	CSF	NC (*n* = 13), iRBD (*n* = 23), PDND (*n* = 21), PDD (*n* = 20)	ELISA	CSF o-α-Syn higher in PDND vs. iRBD (*p* = 0.043), and in PDD vs. NC (*p* = 0.043) and vs. iRBD (*p* = 0.001)
Majbour et al. (24)	2020	CSF	NC (*n* = 43), sPD (*n* = 60), asymptomatic LRRK2 mutation carriers (*n* = 51), symptomatic LRRK2 mutation carriers (*n* = 23)	ELISA	sPD (*p* < 0.001) and asymptomatic LRRK2 (*p* < 0.01) groups had higher CSF o-α-syn levels than NC
Park et al. (21)	2011	CSF, Plasma	NC (*n* = 29), drug-naïve PD (*n* = 23)	ELISA	CSF o-α-Syn higher in drug-naïve PD vs. NC (*p* = 0.001), plasma o-α-Syn no difference in drug-naïve PD vs. NC (*p* = 0.156)
Foulds et al. (22)	2011	Plasma	NC (*n* = 30), PD (*n* = 32)	ELISA	Plasma o-α-Syn no difference in PD vs. NC (*p* = 0.221)
Pchelina et al. (23)	2017	Plasma	NC (*n* = 27), sPD (*n* = 23), GBA-PD (*n* = 22)	ELISA	Plasma o-α-Syn higher in GBA-PD vs. sPD (*p* = 0.002), and in GBA-PD vs. NC (*p* < 0.0001)
Chen et al. (25)	2019	Plasma	NC (*n* = 30), early PD* (*n* = 60)	ELISA	Plasma o-α-Syn higher in early PD vs. NC (*p* < 0.05)
Zhao et al. (26)	2022	Plasma	NC (*n* = 42), PD (*n* = 79), PDs (*n* = 24)	ELISA	PD (*P* < 0.001) and PDs (*p* < 0.05) groups had higher plasma o-α-syn levels than NC
Angius et al. (27)	2023	Salivary	NC (*n* = 23), PD (*n* = 15)	ELISA	Salivary o-α-Syn higher in PD vs. NC (*p* < 0.05)
Vivacqua et al. (28)	2016	Salivary	NC (*n* = 40), PD (*n* = 60)	ELISA	Salivary o-α-Syn higher in PD vs. NC (*p* < 0.01)

*Early PD was defined as Parkinson’s disease patients with Hoehn-Yahr stage ranging from 1 to 2. o-α-syn, alpha-synuclein oligomers; CSF, cerebrospinal fluid; NC, normal control; iRBD, isolated Rapid Eye Movement Sleep Behavior Disorder; PDND, PD with no dementia; PDD, PD with dementia; sPD, sporadic PD; LRRK2, leucine-rich repeat kinase 2; GBA-PD, PD patients with mutations in the GBA gene; PD, Parkinson’s disease; PDs, Parkinson’s disease syndromes; ELISA, enzyme-linked immunosorbent assay.

Our study further revealed a significant positive correlation between plasma o-α-syn levels and MoCA scores in patients with iRBD after FDR correction. Additionally, patients with cognitive impairment tend to have slightly lower plasma o-α-syn levels compared to cognitively normal patients, although not statistically significant. This suggests that lower plasma levels of α-syn may be associated with the occurrence of cognitive impairment in iRBD patients (31–33). Thus, our findings support the hypothesis that aggregate proteins, commonly associated with PD and Alzheimer’s disease, collectively contribute to the pathogenesis of cognitive impairment in PD. Lower levels of α-syn related proteins detected in patients with cognitive impairment may not reflect actual reduction but rather their interaction with Aβ and tau, impacting their accurate detection (34). Interestingly, a cross-sectional study involving 80 PD patients and 34 controls found significantly elevated plasma α-syn levels in PD patients compared to the control group. Additionally, plasma α-syn levels in PD patients with dementia were significantly higher than those in PD patients with mild cognitive impairment or cognitive normalcy, showing a negative correlation with MMSE scores (35). Hall et al. (36) demonstrated that higher levels of CSF α-syn were associated with a rapid progression of motor symptoms and cognitive decline in PD within 2 years. The variations in results of above studies may stem from differences in cognitive assessment methods, sample types utilized, and detection methods employed. Hence, these findings should be interpreted cautiously. Further research is warranted to investigate the mechanisms and implications of this hypothesis.

Biomarkers for diagnosis and disease progression obtained from blood is an attractive option for synucleinopathies, given the infeasibility of tissue biopsy from the central nervous system. However, longitudinal studies on predictive biomarkers for synucleinopathies based on α-syn-related proteins have not produced promising results (37). In this longitudinal study, to our knowledge, we explored for the first time whether plasma o-α-syn could assess the risk of disease progression to synucleinopathies in iRBD patients. After a median follow-up of 5.83 years in iRBD patients, stratified analysis based on baseline plasma o-α-syn levels revealed that individuals with lower levels may have a higher risk of disease phenoconversion, while the *p*-value from the Log-rank test only reached borderline significance. This phenomenon is consistent with a trend of slightly lower CSF α-syn levels observed in iRBD patients and a significant reduction in CSF α-syn levels seen in PD patients (30, 38). Although the underlying mechanism remains unclear, this may involve intracellular aggregation of α-syn in the brain. Hence, larger-scale longitudinal studies are warranted in the future to elucidate the predictive value of plasma o-α-syn in iRBD conversion and their potential role in disease management. Moreover, alterations in circulating microRNA (39) and serum neurofilament light chain (40) levels have been validated in some longitudinal studies of iRBD as predictive indicators for conversion. Therefore, future research should focus on integrating various synuclein and non-synuclein biomarkers to capture disease signals comprehensively during the prodromal phase of synucleinopathies, aiming to enhance the reliability and clinical value of current detection methods.

It is important to acknowledge the limitations of our study. First, our sample size was relatively small, especially for longitudinal analysis. Meanwhile, some patients are newly enrolled and still have a short follow-up time. After an average follow-up of 5.83 years, only 26 (36.11%) patients transitioned from iRBD to α-synucleinopathies. Therefore, it is necessary to extend the follow-up time on a larger scale cohort basis to confirm the reliability of our results. Second, we did not assess o-α-syn levels in CSF in our subjects to explore consistency in the changes between the central nervous system and peripheral blood. Furthermore, the ELISA technique used to detect o-α-syn may have influenced the results due to reported variability in outcomes depending on the type of antibodies used in the kit and the sample type. Thus, our results should be interpreted with caution. Future studies should work toward standardizing ELISA protocols across different studies to enhance comparability in the field (30). Finally, hemolysis-induced contamination is an inevitable issue that may impede the accurate evaluation of alterations in plasma α-syn levels (41). Although we employed a standardized blood processing procedure and strictly controlled the time interval between blood collection and plasma separation to reduce interference from cell lysis, we did not measure plasma hemoglobin levels. Some studies have indicated that red blood cell hemolysis affects total plasma α-syn levels but not oligomeric plasma α-syn levels (23), so this potential influence is unlikely to be significant in our study.

In conclusion, our study suggests that plasma o-α-syn levels exhibits limited diagnostic efficacy for iRBD patients. However, the correlation between plasma o-α-syn levels and cognitive decline emphasizes their potential role in elucidating the pathogenesis of α-synucleinopathies. Moreover, a baseline lower levels of plasma o-α-syn in iRBD patients may be associated with a higher risk of conversion. However, the reliability of this conclusion requires further research for validation, and the underlying mechanism should be explored to uncover the complex interactions between α-syn pathology and disease progression in iRBD.

## Data Availability

The raw data supporting the conclusions of this article will be made available by the authors, without undue reservation.
